# COVID-19 Information Overload and Cyber Aggression during the Pandemic Lockdown: The Mediating Role of Depression/Anxiety and the Moderating Role of Confucian Responsibility Thinking

**DOI:** 10.3390/ijerph19031540

**Published:** 2022-01-29

**Authors:** Qiong Wang, Xiao Luo, Ruilin Tu, Tao Xiao, Wei Hu

**Affiliations:** 1 School of Education, Zhengzhou University, Zhengzhou 450001, China; wangq@zzu.edu.cn (Q.W.); 202012122012108@gs.zzu.edu.cn (X.L.); 202012122012109@gs.zzu.edu.cn (R.T.); 2 Department of Psychology, School of Philosophy, Wuhan University, Wuhan 430072, China; xtwolo@163.com; 3 Information Engineering University, Zhengzhou 450001, China

**Keywords:** COVID-19 information overload, cyber aggression, Confucian responsibility thinking, anxiety, depression

## Abstract

Many countries adopted lockdown measures to curb the spread of the outbreak in 2020, while information about COVID-19 has dominated various media outlets, which has led to information overload for people. However, previous research has mainly focused on cancer information overload and the corresponding consequence, and failed to examine its adverse effects in the context of major public health events. Based on the Frustrate Aggression Theory and the Scapegoat Theory, the present study established a moderated mediation model to investigate the emotional and behavioral outcomes of COVID-19 information overload. The mediating role of depression/anxiety in the association between COVID-19 information overload and cyber aggression, as well as the moderating role of Confucian responsibility thinking, were tested. This model was examined with 1005 Chinese people (mean age = 26.91 years, *SD* = 9.94) during the COVID-19 outbreak. Mediation analyses revealed that COVID-19 information overload was positively related to cyber aggression, depression, and anxiety, parallelly and partially mediated this relationship. Moderated mediation analyses further indicated that Confucian responsibility thinking not only moderated the direct link between COVID-19 information overload and cyber aggression, with the effect being significant only for people with a low level of Confucian responsibility thinking, but also moderated the relationship between COVID-19 information overload and depression/anxiety respectively, with the associations being much more potent for individuals with low levels of Confucian responsibility thinking. These findings have the potential to inform the development of prevention and intervention programs designed to reduce the negative emotions and cyber aggression associated with information overload in public health events.

## 1. Introduction

Since the first outbreak in December 2019, coronavirus (COVID-19) has affected and is still affecting large numbers of people around the world. Many countries adopted lockdown measures to curb the spread of coronavirus in 2020. The outbreak has severely impacted the information environment, as well as the daily lives of individuals around the world, with information about COVID-19 dominating all online and offline media [1]. People have an ingrained need to access to and share information in a crisis [2], in order to reduce uncertainty and negative feelings about it. However, enormous amounts of information about the epidemic may lead to information overload for individuals [3]. Information overload is a state in which information exceeds the range that an individual can accept, process, and cope with [4]. A multitude of studies have documented that information overload may lead to negative impacts on mental and physical health, such as perceived stress, loss of control, depression, and life dissatisfaction [5,6,7].

According to the Frustrate Aggression Theory, which holds that stress and aggressive behavior are closely related [8,9], people will feel frustrated and have strong aggressive tendencies in a stressed situation. The epidemic situation is a strong social stress, in which people are more aggressive [10,11]. The stay-at-home quarantine order during the outbreak in China requested people to stay at home for a long time; it is not an overstatement that people use the Internet to receive information, to contact others, and release their emotions more often than ever before [1,12]. People’s aggressive behavior may be switched from offline to online in such a situation, with studies showing a significant increase in cyber aggression during the pandemic [13,14]. It might be the influence of COVID-19 information overload. Although previous research has explored that information overload’s consequences mainly focus on negative emotions, health information avoid, cancer fear et al. [5,7,15], the present study aimed to investigate the relationship between the COVID-19 information overload and cyber aggression. In order to know more about the “how” and “why” mechanism behind this association, we explored a moderated mediation model in the context of the early COVID-19 outbreak in China. Thus, the current study utilized a Chinese sample to determine whether negative emotions (e.g., depression and anxiety) would mediate the relationship between COVID-19 information overload and cyber aggression, and whether the mediating process could be moderated by individual factors (e.g., Confucian responsibility thinking).

### 1.1. COVID-19 Information Overload and Cyber Aggression

Cyber aggression, as a kind of aggressive behavior, refers to behaviors that make a target feel offended, which are derogatory, harmful, or unwanted, through the use of digital/electronic media [16,17]. Previous work has shown that stressful situations will lead to cyber aggression [18], while information overload can increase the perception of pressure [19,20]. When faced with the turbulent “information flow”, people are more likely to have negative information bias, and pay attention to the information with a negative bias of threat or loss [21], thus further increasing the stress level.

Meanwhile, although humans have the ability to regulate themselves, ego depletion will appear after experiencing some activities that require self-control resources, which may increase the probability of verbal attack and aggression [22,23,24]. People need to make more efforts to cope with current tasks when they are information-overloaded [25], and long-lasting cognitive efforts will lead to self-exhaustion [26]. Bawden and Robinson [27] have found that information overload can lead to loss of control, and result in feelings of being overwhelmed. According to the Ego Depletion Theory [22,23], many behavioral and social problems stem from persistent lapses in self-control, such as cyber aggression. Thus, it is reasonable to assume that COVID-19 information overload will increase people’s cyber aggression levels.

**Hypothesis** **1** **(H1).** *COVID-19 information overload will positively correlate with cyber aggression*.

### 1.2. The Mediating Role of Depression and Anxiety

Negative emotions may play mediating roles in COVID-19 information overload and cyber aggression. First, COVID-19 information overload may induce the increase of negative emotions. Negative emotional contagion occurs widely in the network platform through information transmission [28]. Previous research has shown that social medias such as Facebook and Twitter are typical emotional contagion platforms, where people are easily infected by the emotions of others and become part of online events that trigger various emotional responses [29,30]. During the outbreak, people have been accessing COVID-19 information and communicating with others via the Internet on a daily basis, and are likely to be drawn into the emotions spread online, thus exacerbating their own negative emotions, since people are more likely to pay more attention to negative information in the epidemic, because of negative information bias [21]. Previous studies have also proved that information overload will induce more negative emotions, such as anger, anxiety, and depression [31]. This phenomenon may be more prominent during stay-at-home quarantine.

Second, negative emotions can lead to cyber aggression. Previous studies have reported that negative emotions can lead to violence, aggression, and other offline aggressive behaviors [24,32,33]. The Scapegoat Theory holds that people need a “scapegoat” as a vent when they are unable to cope with the causes of their negative emotions [34]. Facing with the increasing number of infections and out of control of the COVID-19, people may need a scapegoat that can be attacked when they cannot explain the natural disasters and man-made catastrophes that they encounter. As a result, people may vent their negative emotions through cyber aggression, since it is anonymous and easy to disguise. What’s more, negative emotions can lead to higher ego depletion compared to positive emotions [35,36]. Previously, research has shown that individuals need to use their limited self-resources to cope with negative emotions when the external environment is inconsistent with their expectations, which leads to ego depletion [37], and in turn, leads to immoral or risky behavior [38,39], such as cyber aggression.

Depression and anxiety are the typical negative emotions of the people in the outbreak of COVID-19 [40,41]. People may be depressed and anxious because of the uncertainty of the outbreak and fear of the serious consequences of the outbreak when they were overloaded with COVID-19 information [1,6,7,11]. Besides, a large number of studies have shown that depression is significantly associated with aggressive behavior [42,43], as well as anxiety [43,44]. Combined, it is possible that COVID-19 information overload can be positively associated with depression and anxiety, which in turn, can increase people’s cyber aggression. Based on the literature reviewed above, we put forward the following hypothesis:

**Hypothesis** **2** **(H2).** *Depression and anxiety will mediate, in parallel, the association between COVID-19 information overload and cyber aggression*.

### 1.3. The Moderating Role of Confucian Responsibility Thinking

While COVID-19 information overload and triggered negative emotions may drive cyber aggression during the outbreak, people are able to control and alter different aspects of emotional processing. People from different cultural backgrounds have different psychological and behavioral characteristics. Chinese people’s coping thinking in the COVID-19 outbreak reflects the unique Confucian characteristics, since Confucian thought is the mainstream of Chinese social culture [45]. Confucian responsibility thinking, as an important part of Confucian coping thinking, refers to the thinking that people should be born with social responsibility, advocates “taking the world as one’s own duty” and “restrain yourself and yield to others”, which emphasize the consciousness of concerns about the state and people when dealing with difficulties [46,47]. During the outbreak of the COVID-19, Can Chinese people’s unique way of thinking reduce the public’s negative emotions and cyber aggression? Chinese traditional culture provides a new research perspective.

Chinese people are deeply influenced by Confucianism, and they often use Confucian coping, either consciously or unconsciously, to cope with adversity and stress [48]. As a coping style, Confucian responsibility thinking emphasizes the responsibility for others and self-restraint under pressure [49]. Individuals with high level of Confucian responsibility thinking are stricter with themselves, give others more convenience, and comply with others’ needs [47]. Thus, they might be less likely to perpetrate aggressive behaviors to other people, especially during the outbreak of COVID-19, a period when most people were panicked and upset. It is reasonable to assume that Confucian responsibility thinking can buffer the relationship between COVID-19 information overload and cyber aggression.

Furthermore, Confucian responsibility thinking can positively predict individuals’ mental health [50], which can have an intergenerational transmission effect in this association [51]. Researchers have found that Confucian responsibility thinking is positive related to the individual powers of resilience and life satisfaction [46,50], which may help people to be less pessimistic when they were COVID-19-information-overloaded. A large number of studies have consistently found a negative effect of Confucian responsibility thinking on anxiety and depression among Chinese people of different ages [46,48,50,52]. Therefore, we hypothesized that Confucian responsibility thinking can buffer the negative emotions (depression and anxiety) caused by information overload in the COVID-19 outbreak. Based on the literature reviewed above, we put forward the following hypotheses:

**Hypothesis** **3** **(H3).** *Confucian responsibility thinking will buffer the direct association between COVID-19 information overload and cyber aggression*.

**Hypothesis** **4** **(H4).** *Confucian responsibility thinking will moderate the indirect relations between COVID-19 information overload and cyber aggression by buffering the associations between COVID-19 information overload and depression/anxiety*.

### 1.4. The Present Study

**Figure 1:**
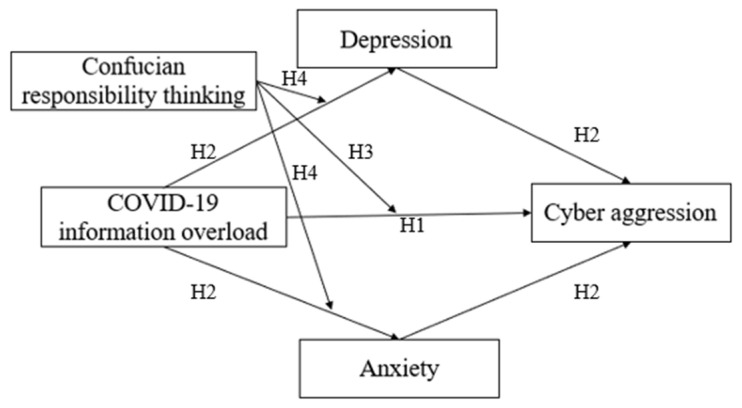
The proposed, moderated double-mediation model.

In this study, we tested a moderated mediation model of the association among COVID-19 information overload, depression, anxiety, Confucian responsibility thinking, and cyber aggression among Chinese people. The aims of this study were threefold: first is to test whether COVID-19 information overload would positively associate with cyber aggression; second is to test whether depression and anxiety would parallelly mediate the relation between COVID-19 information overload and cyber aggression; third is to test the moderating role of Confucian responsibility thinking in the direct and indirect relations between COVID-19 information overload and cyber aggression, through depression and anxiety as mediators. Taken together, these three research questions established a moderated mediation model (Figure 1).

## 2. Method

### 2.1. Participants

Our sample was recruited from Henan province, China. A total of 1027 questionnaires were collected through our survey during the COVID-19 outbreak (14 to 20 February 2020). After discarding the invalid questionnaires, 1005 valid questionnaires were obtained, yielding a valid response rate of 97.86%. The participants consisted of 463 males (46%) and 542 females (54%). The mean age of the participants was 26.91 years (*SD* = 9.94, range = 18–60 years).

### 2.2. Measures

#### 2.2.1. COVID-19 Information Overload

COVID-19 information overload was assessed by the Chinese version of the Information Overload Scale [53]. This scale comprises of 3 items, and we have added some information to make it more suitable for our study (“I am distracted by the excessive amount of epidemic information available to me”, “I find that I am overwhelmed by the amount of epidemic information I have to process on a daily basis”, and “Usually, my problem is with too much epidemic information to synthesize instead of not having enough information to make decisions”). The participants answered the items using a nine-point scale (1 = Strongly Disagree; 9 = Strongly Agree). The higher the average score, the higher the level of information overload. In this study, the Cronbach’s α for the information overload was 0.78.

#### 2.2.2. Cyber Aggression

Cyber aggression was assessed using the Chinese version of the Scale for Internet Deviance (SID) [54]. It assesses four factors related to cyber aggression, including hostility, aggression, conflict, and irritability. Participants were asked to answer 20 items (e.g., “Whenever conflicts arise with someone on the Internet, I send them some offensive symbols/pictures.”) on a 5-point scale (1 = “Never” to 5 = “Always”). This scale has been used in Chinese adolescent and young adults with good reliability and validity [54,55]. A higher average score represented a higher level of cyber aggression. In this study, the Cronbach’s α for cyber aggression was 0.96.

#### 2.2.3. Depression

The Chinese version of the Patient-Reported Outcomes Measurement Information System (PROMIS) depression scale, developed by Primack et al. [6], was used to measure the frequency of participants’ experience of feeling hopeless, worthless, helpless, or depressed during the past seven days. It included three items, and each item was scored on a 5-point Likert scale ranging from 1 = “Never” to 5 = “Always”. Higher scores represent greater depression symptom severity. In the present study, the Cronbach’s α for depression was 0.85.

#### 2.2.4. Anxiety

We assessed anxiety symptoms using the Chinese version of the 4-item PROMIS anxiety scale [6]. Participants were asked how frequently they had experienced the following anxious symptoms in the past seven days: “I felt fearful,” “I felt it was hard to focus on anything other than my anxiety,” “My worries overwhelmed me,” and “I felt uneasy”. All the items were in the form of a 5-point Likert scale, ranging from 1 = “Never” to 5 = “Always”. Higher scores represent greater anxiety symptom severity. In this study, the Cronbach’s α was 0.85.

#### 2.2.5. Confucian Responsibility Thinking

Items for measuring Confucian responsibility thinking were adopted from the Confucian Coping Scale [50]. This Chinese questionnaire consists of five items (e.g., “People naturally assume social responsibilities.” and “Even at my worst failure, I felt hopeful for the future.”) and were in the form of a 5-point Likert scale, which ranged from 1 (Strongly Disagree) to 5 (Strongly Agree). Responses were averaged across the eight items, with higher scores representing higher levels of Confucian responsibility thinking. In this study, Cronbach’s α for the Confucian responsibility thinking was 0.80.

### 2.3. Procedure

Using convenient sampling, participants were recruited through a Chinese online survey platform (https://www.wjx.cn/, accessed on 19 January 2021) during the pandemic outbreak from 14 to 20 February 2020. On the first page of the survey, an information sheet describing the study objectives and procedures was presented to participants, and the principle of anonymity and confidentiality was emphasized to reduce the participants’ concern. All the participants voluntarily participated in this study, and they were free to withdraw from this study at any time. Participants completed questionnaires regarding demographics, COVID-19 information overload, anxiety, depression, cyber aggression, and Confucian responsibility thinking. It took approximately 15 min to complete all questionnaires; participants received thanks and a random fee of about CNY 8. All finished questionnaires were automatically sent back to and stored by the platform, which were available to be transformed into downloadable formats. This investigation was approved by the Ethics Committee of the first author’s institution.

### 2.4. Statistical Analyses

To organize and analyze the data, we used SPSS 25.0 (IBM, Armonk, NY, USA) and the Hayes SPSS macro program PROCESS [56]. First, the descriptive information and correlation matrix was calculated. Second, we selected Model 4 to analyze the mediating effect of depression and anxiety on the relationship between COVID-19 information overload and cyber aggression. Then, we used Model 8 to test the moderated mediation model to examine the moderating role of Confucian responsibility thinking. Gender and age were controlled in all analyses. All regression coefficients were tested by the bias-corrected percentile Bootstrap method. The theoretical hypothesis model was tested by estimating the 95% confidence interval (CI) for mediation and moderating effects, with 5000 resampled samples [56]. Confidence intervals that did not include zero indicated statistically significant effects. Before formal data processing, all variables were standardized.

## 3. Results

### 3.1. Preliminary Analyses

Table 1 presents the means, standard deviations, and correlation coefficients among variables. As expected, COVID-19 information overload was positively correlated with depression (*r* = 0.34, *p*< 0.001), anxiety (*r* = 0.38, *p* < 0.001), and cyber aggression (*r* = 0.25, *p* < 0.001). Depression and anxiety were positively correlated with cyber aggression (*r* = 0.52, *p* < 0.001; *r* = 0.49, *p* < 0.001). The results provided initial evidence for the hypotheses.

### 3.2. Testing for the Mediation Effect

After controlling for the variables of gender and age, a double-mediating analysis was conducted to explore the mediating effects of depression and anxiety using Model 4 of the PROCESS macro. The results are shown in Table 2. Model 1 of Table 2 shows that the positive predictive effect of COVID-19 information overload on depression was significant (*β* = 0.33, *t* = 11.03, *p* < 0.001), while depression had a positive predictive effect on cyber aggression (Model 3, *β* = 0.33, *t* = 6.16, *p* < 0.001). Model 2 of Table 2 shows that COVID-19 information overload positively predicts anxiety (*β* = 0.37, *t* = 12.53, *p* < 0.001), which, in turn, was also positively related to cyber aggression (Model 3, *β* = 0.18, *t* = 3.32, *p* < 0.001). Moreover, when mediating variables were added, the direct predictive effect of COVID-19 information overload on cyber aggression was still significant, as shown in Model 3 of Table 2 (*β* = 0.08, *t* = 2.68, *p* < 0.01).

Table 3 shows the results of the bootstrap analysis. The upper and lower bounds of the bootstrapped 95% CI did not include 0, indicating that the direct effect of COVID-19 information overload on cyber aggression and the mediating effect of depression and anxiety were all significant. The total indirect effect accounted for 69.84% of the total effect. Specifically, the mediation effect of depression was 0.109, accounting for 43.25% of the total effect, while the mediation effect of anxiety was 0.067, accounting for 26.59% of the total effect. Thus, depression and anxiety played a parallel partial mediating role in the relationship between COVID-19 information overload and cyber aggression; Hypothesis 2 was therefore supported.

### 3.3. Testing for the Moderated Mediation

**Figure 2:**
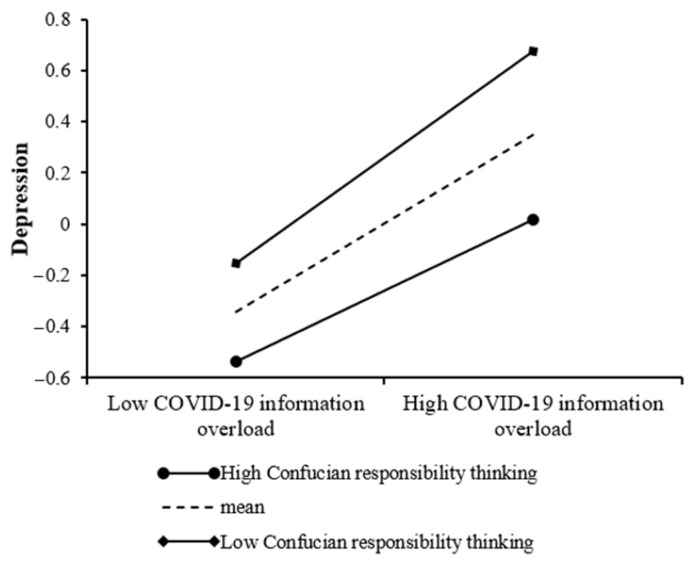
Confucian responsibility thinking moderates the relationship between COVID-19 information overload and depression.

To test the moderated mediation model, we used the PROCESS macro Model 8 developed by Hayes (2013). The results are shown in Table 4. First, as shown in Model 1 of Table 4, the effect of COVID-19 information overload on depression was significant (*β* = 0.35, *p* < 0.001), and this effect was moderated by Confucian responsibility thinking (*β* = −0.07, *p* < 0.05). To describe the moderating effect, this study presented COVID-19 information overload on depression at different levels of Confucian responsibility thinking (*M*, *M* − 1 *SD* and *M* + 1 *SD*) (Figure 2). COVID-19 information overload significantly predicted depression in high-level Confucian responsibility thinking and low-level Confucian responsibility thinking, but the predictive function of COVID-19 information overload on depression was much stronger for individuals with low levels of Confucian responsibility thinking (*β*_simple_ = 0.41, *SE* = 0.04, *p* < 0.001, 95% CI = [0.33, 0.50]) than for individuals with high levels of Confucian responsibility thinking (*β*_simple_ = 0.28, *SE* = 0.04, *p* < 0.001, 95% CI = [0.20, 0.35]).

**Figure 3:**
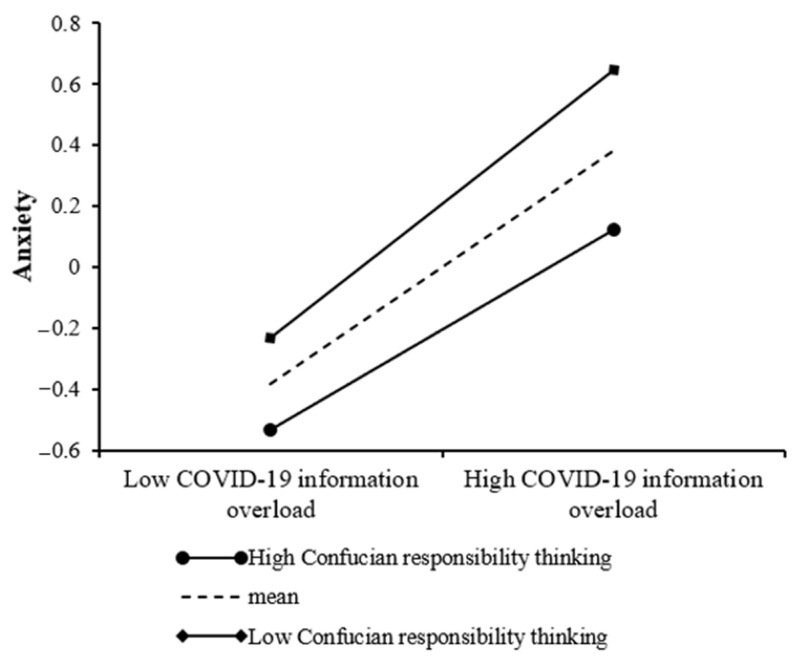
Confucian responsibility thinking moderates the relationship between COVID-19 information overload and anxiety.

Model 2 of Table 4 indicated that the effect of COVID-19 information overload on anxiety was significant (*β* = 0.38, *p* < 0.001), and the interaction of COVID-19 information overload and Confucian responsibility thinking also showed a significant predictive effect on anxiety (*β* = −0.06, *p* < 0.05). That is, Confucian responsibility thinking moderated the relationship between COVID-19 information overload and anxiety. Specifically, the simple slope test in Figure 3 indicates that the association between COVID-19 information overload and anxiety is significantly stronger for participants with low levels of Confucian responsibility thinking (*β*_simple_ = 0.44, *SE* = 0.04, *p* < 0.001, 95% CI = [0.36, 0.52]), whereas this positive association is much weaker for participants with high levels of Confucian responsibility thinking (*β*_simple_ = 0.33, *SE* = 0.04, *p* < 0.001, 95% CI = [0.25, 0.40]), indicating Confucian responsibility thinking’s buffering role in this relationship.

**Figure 4:**
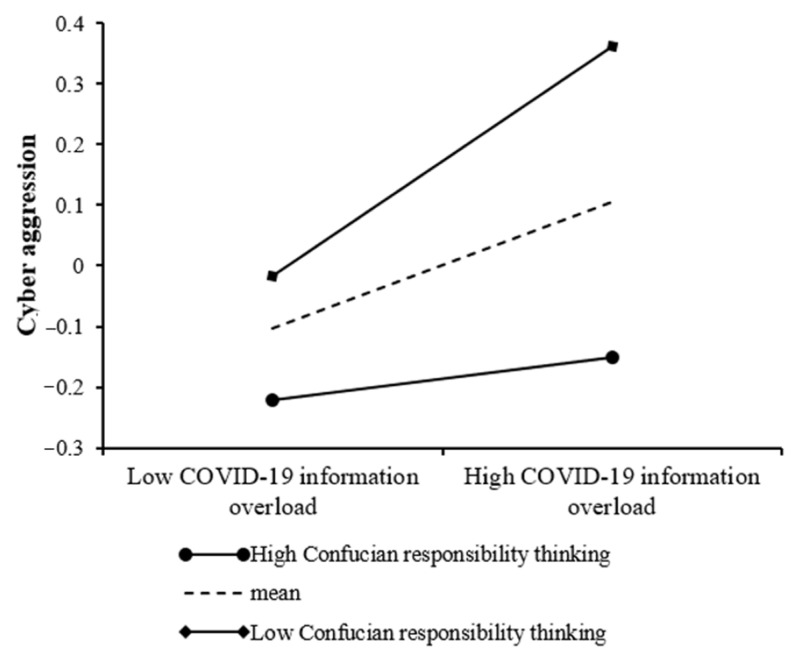
Confucian responsibility thinking moderates the direct effect of COVID-19 information overload on cyber aggression.

As shown in Model 3 of Table 4, the direct effect of COVID-19 information overload on cyber aggression was significant (*β* = 0.10, *p* < 0.001), and this effect was moderated by Confucian responsibility thinking (*β* = −0.07, *p* < 0.01). Simple slope tests in Figure 4 demonstrated that, for participants with lower levels of Confucian responsibility thinking, higher levels of COVID-19 information overload were associated with higher levels of cyber aggression (*β*_simple_ = 0.17, *SE* = 0.04, *p* < 0.001, 95% CI = [0.10, 0.25]). However, for participants with high levels of Confucian responsibility thinking; the association between COVID-19 information overload and cyber aggression was non-significant (*β*_simple_ = 0.04, *SE* = 0.04, *p* = 0.31, 95% CI = [−0.03, 0.10]). Therefore, Hypothesis 3 was supported.

The bias-corrected percentile bootstrap analyses further verified that the indirect effect of COVID-19 information overload on cyber aggression through depression and anxiety is moderated by Confucian responsibility thinking. The indirect relationship between COVID-19 information overload on cyber aggression through depression is significantly stronger for participants with low levels of Confucian responsibility thinking (*β* = 0.11, *SE* = 0.03, 95% CI [0.05, 0.17]), whereas this indirect relationship is much weaker for participants with high levels of Confucian responsibility thinking (*β* = 0.07, *SE* = 0.02, 95% CI [0.04, 0.12]). At the same time, the effect of the path “COVID-19 information overload→anxiety→cyber aggression” was stronger for participants with low levels of Confucian responsibility thinking (*β* = 0.08, *SE* = 0.03, 95% CI [0.03, 0.14]) than participants with high levels of Confucian responsibility thinking (*β* = 0.06, *SE* = 0.03, 95% CI [0.02, 0.11]). Therefore, Hypothesis 4 was supported.

## 4. Discussion

During the outbreak, people cannot help but frequently browse epidemic information in home quarantine, which leads to COVID-19 information overload. Previous research exploring information overload mainly focused on cancer information and the corresponding results, such as anxiety, cancer, fear, and more [57]. Although some studies start to explore the behavioral consequences of information overload, such as health information avoidance [15] and lack of compliance with recommended behaviors [58], and no studies have examined the externalizing problem of it. Thus, we formulated a moderated mediation model to test how COVID-19 information overload works on cyber aggression, and whether all people are equally influenced by it. Consistent with our hypotheses, our findings indicated that COVID-19 information overload was significantly and positively associated with cyber aggression among Chinese people, while depression and anxiety partially mediated this relationship. Furthermore, Confucian responsibility thinking moderated the direct and indirect relationship between COVID-19 information overload and cyber aggression. The results have certain theoretical and practical implications for deepening our understanding of the relationship between information overload, emotions, and behaviors.

### 4.1. COVID-19 Information Overload and Cyber Aggression

We first confirmed a positive relationship between COVID-19 Information overload and cyber aggression, which supported Hypothesis 1. This result was consistent with the Frustration–Aggression Theory [8], which indicates that aggressive behavior may be a psychological defense triggered by the external stress situation in order to relieve the inner pressure [9]. The pandemic, as a special case of major public health events, is a strong social stress situation, and the COVID-19 information overload makes it even worse by increasing the stress people perceived [5,59]. Compared with violence and attack in real life, cyber aggression might become a new way for people to vent their emotions and stresses in COVID-19 epidemic, since it is more covert and less costly [13].

Cognitively speaking, the Dual Process of Thinking Theory indicates that there are system 1 (intuitive, high capacity) and system 2 (reflective, low capacity) processes cognitively [60,61]. The system 2 process involves being in a loaded state, wherein people are “cognitively busy”: overloaded with information about the pandemic, leaving system 1 “free”, which then probably leads to selfish/impulsive and not well thought out choices, such as aggressive behaviors. As people have to use more cognitive resources to process the large amount of mostly negative information about the epidemic [27], their self-control ability might be reduced [39], which may increase the likelihood of aggressive behaviors [24,38], especially on the Internet; the place that most people used to communicate with others during the lockdown period (except for family members).

### 4.2. The Mediating Effects of Depression and Anxiety

Consistent with our hypothesis 2, this study showed that COVID-19 information overload could be positively related to cyber aggression; depression and anxiety partially mediated this relationship. Thus, both depression and anxiety could serve as one of the “bridges” that link COVID-19 information overload and cyber aggression, which support the Scapegoat Theory [34]. According to this theory, this result can be explained that when people cannot deal with the cause of their negative emotions, they look for a “scapegoat” to vent their frustrations.

On the one hand, our findings provide evidence for the notion that COVID-19 information overload can be positively related to depression and anxiety. It is in line with previous research studies, which have shown that information overload may increase the likelihood of developing negative emotions [1,7,31]. This may be due to people’s feeling of uncertainty, fear, and dread of the new coronavirus information when they are kept in isolation and quarantine [41,62]. People were easily infected by the emotions of others because of the emotional contagion on social medias [29], while the negative information bias makes people pay more attention to negative information about COVID-19 [21]. Thus, information overload in the epidemic means that people are immersed in a flood of negative information and inevitably suffer from emotional contagion, resulting in a variety of negative emotions, such as depression and anxiety.

On the other hand, this study revealed that individuals with more depressive and anxious emotions were more likely to perpetrate cyber aggression. Previous studies have found that cyberbullying can lead to individual depression and anxiety [11,63], which, in turn, are the result variables of cyberbullying victimization [64,65], while others studies have found the predictive role of depression and anxiety to aggression [42,44,66]. These variables have a reciprocal relationship with each other [43,67]. Our study extends previous research by demonstrating that depression and anxiety may trigger aggressive behaviors online in a COVID-19 information overloaded state, which may help people to understand this relationship further. In addition, although previous studies have suggested that depression and anxiety have different correlations with aggressive behavior [68], the results of the present study showed that depression and anxiety play a similar role in the relationship between information overload and cyber aggression. This may be due to the high correlation between anxiety and depression during the COVID-19 outbreak [69].

### 4.3. The Moderating Role of Confucian Responsibility Thinking

Our results also showed that Confucian responsibility thinking buffered the relationship between COVID-19 information overload and cyber aggression, as well as the relationship between COVID-19 information overload and depression/anxiety, supporting hypothesis 3 and hypothesis 4. First, the direct relationship between COVID-19 information overload and cyber aggression was significant for individuals with a low level of Confucian responsibility thinking, but not significant for individuals with a high level of Confucian responsibility thinking. This might be because individuals with Confucian responsibility thinking are more likely to hold a self-restraint state under pressure, such as COVID-19 information overload [49]. “Cultivate one’s morality, improve one’s family, run a country and make the world peaceful”, Confucianism emphasizes that gentlemen should not forget their own responsibility, regardless of what circumstances they are in, so they will choose to restrain themselves and do what they should do, even when they are impacted by excessive negative information [49]. When they access a lot of COVID-19 information from various media, people with a Confucian mindset of responsibility are more demanding of themselves, and are willing to make life more convenient for others [47], so they are less likely to harass or bully others on the Internet.

Second, the results were consistent with the hypothesis that Confucian responsibility thinking would buffer the relationship between COVID-19 information overload and negative emotions (depression and anxiety), as the association between psychological maltreatment and depression/anxiety was stronger for adolescents with low levels of Confucian responsibility thinking. Echoing previous studies, these results supported the notion that Confucian responsibility thinking can be a protective factor for anxiety and depression [48,52]. The reasons may be as follows. Confucian responsibility thinking, as an attitude and coping style in the context of Chinese culture, helps people to have more sense of life satisfaction [46], which obviously lessens depression [70,71], and is highly correlated with anxiety [72]. Confucian responsibility thinking emphasizes the responsibility of individuals in difficult situations [47,49], and changes people’s cognition in epidemic situations from a positive perspective [45]. For example, Chinese people take cutting off the infection by staying at home as their responsibility, as well as the greatest contribution to society, and the country for every ordinary person in the fight against the epidemic. Thus, they can accept and strictly follow the home quarantine order, and decrease the negative feelings of people for limited mobility in the epidemic. In addition, since Confucian responsibility thinking is positively related to individuals’ powers of resilience [50], people with a high level of Confucian responsibility thinking may adjust themselves appropriately when they are overloaded with COVID-19 information. Therefore, they are less likely to be depressed and anxious, or to have less of it.

### 4.4. Limitations and Implications

There are some limitations to this study that need further investigation in future research. First, this work is a cross-sectional designed study, which cannot deduce causality from the results. Experimental or longitudinal studies should be conducted to confirm the casual assumptions in this study. Second, adolescents’ self-report measures may have social desirability effects, because people are prone to underestimate their cyberaggression perpetration or over-evaluate the Confucian responsibility thinking. Multiple informants should be encouraged in future research to replicate our results. Third, although this study used a Chinese sample with a relatively wide age range, it still cannot represent the general population perfectly; future studies can benefit from the use of a larger and more representative sample. Fourth, the present study explored the model in the special context of COVID-19; future studies can test the relationship between information overload and cyber aggression in other circumstances. Furthermore, since this study takes Confucian responsibility thinking, a coping strategy often seen from the Chinese, as a buffer, it would also be helpful to examine other important underlying mechanisms in the relationship between information overload and cyber aggression, such as self-compassion, which is well accepted in Western culture.

Despite these limitations, the results of this study do have important theoretical and practical implications. Specifically, the present study constructed an integrated model to examine the unique and interactive effects of COVID-19 information overload, depression, anxiety, and Confucian responsibility thinking on cyber aggression. To our knowledge, the current study is the first studies to date to clarify the important role of information overload for cyber aggression during a public health event. The results show that COVID-19 information overload serves as an important influencing factor for people’s negative emotions, and subsequently increases their aggressive behaviors online, which verified the Frustrate Aggression Theory and the Scapegoat Theory again. Future research could extend this to the exploration of offline aggressive behaviors that are often observed in activities that violate the rules of outbreak prevention and control. However, Confucian responsibility thinking as a protective factor can significantly reduce the detrimental effects of COVID-19 information overload on individual emotions (i.e., depression and anxiety) and behaviors (i.e., cyber aggression). These results indicate that responsibility thinking, as an attitude and coping style in Chinese culture, has a significant protective effect on the psychological path of both “state-behavior” and “state-emotion”, especially in a health crisis, such as a widespread epidemic outbreak.

Practically, these results also suggest that positive information can be increased when conducting interventions of aggressive behavior in public health events. For example, a series of positive measures for people’s livelihood and security such as food and transportation can be mentioned when issuing a home quarantine order. At the same time, the relevant departments should make the public aware of the negative impact that long-term searching and browsing negative information may have on them by means of education and publicity, and reduce the time spent searching and browsing epidemic information.

## 5. Conclusions

In summary, the current study reveals that information overload is a risk factor for cyber aggression. Besides, the effect is partially, and in parallel, mediated by depression and anxiety. Furthermore, the direct effect of COVID-19 information overload on cyber aggression was significant when Confucian responsibility thinking was low, but not significant when Confucian responsibility thinking was high. The effects of COVID-19 information overload on anxiety and depression were stronger for people with lower levels of Confucian responsibility thinking than for higher. These findings provide a deeper understanding of the emergence and development of negative emotions and cyber aggression during the epidemic, which contributes to prevention and intervention.

## Figures and Tables

**Table 1:** Descriptive statistics and correlations among variables.

Variables	*M*	*SD*	1	2	3	4	5	6	7
1. Gender	-	-	1						
2. Age	26.91	9.94	0.06	1					
3. COVID-19 information overload	4.16	1.83	0.13 ***	−0.09 **	1				
4. Depression	1.73	0.82	0.05	−0.10 **	0.34 ***	1			
5. Anxiety	1.81	0.83	0.07 *	−0.06 *	0.38 ***	0.87 ***	1		
6. Cyber aggression	1.76	0.75	−0.13 ***	−0.19 ***	0.25 ***	0.52 ***	0.49 ***	1	
7. Confucian responsibility thinking	3.73	0.81	0.06	0.15 ***	0.02	−0.24 ***	−0.18 ***	−0.29 ***	1

Note. *N* = 1005. Gender: male = 0, female = 1; * *p* < 0.05, ** *p* < 0.01, *** *p* < 0.001.

**Table 2:** Testing the mediation effect of COVID-19 information overload on cyber aggression.

Predictors	Model 1		Model 2		Model 3	
(IV)	(DV: Depression)		(DV: Anxiety)		(DV: Cyber Aggression)	
	*β*	*t*	*β*	*t*	*β*	*t*
Age	−0.07	−2.38 *	−0.03	−0.99	−0.13	−4.86 ***
Gender	0.02	0.36	0.04	0.70	−0.31	−5.91 ***
COVID-19 Information overload	0.33	11.03 ***	0.37	12.53 ***	0.08	2.68 **
Depression					0.33	6.16 ***
Anxiety					0.18	3.32 ***
*R* ^2^	0.35		0.38		0.57	
*F*	45.58 ***		55.77 ***		94.26 ***	

Note: *N* = 1005. IV, independent variable; DV, dependent variable. Gender: male = 0, female = 1. * *p* < 0.05, ** *p* < 0.01, *** *p* < 0.001.

**Table 3:** Bootstrap analysis of mediation effects.

Effect	Effect Size	SE	Percentage of Total Effects	95% CI	
				Lower	Upper
Total effect	0.252	0.030	100%	0.192	0.311
Direct effect	0.076	0.028	30.16%	0.020	0.132
Total indirect effect	0.176	0.021	69.84%	0.138	0.218
COVID-19 Information overload→depression→cyber aggression	0.109	0.024	43.25%	0.064	0.158
COVID-19 Information overload→anxiety→cyber aggression	0.067	0.024	26.59%	0.019	0.116

Note. *N* = 1005. Bootstrap = 5000.

**Table 4:** Testing the moderated mediation effect of COVID-19 information overload on cyber aggression.

Predictors	Model 1		Model 2		Model 3	
(IV)	(DV: Depression)		(DV: Anxiety)		(DV: Cyber Aggression)	
	*β*	*t*	*β*	*t*	*β*	*t*
Age	−0.03	−1.13	<0.01	−0.03	−0.11	−4.04 ***
Gender	0.03	0.58	0.05	0.89	−0.30	−5.88 ***
COVID-19 Information overload	0.35	11.88 ***	0.38	13.15 ***	0.10	3.71 ***
CRT	−0.26	−8.77 ***	−0.21	−6.92 ***	−0.19	−6.82 ***
COVID-19 Information overload × CRT	−0.07	−2.51 *	−0.06	−2.06 *	−0.07	−2.82 **
Depression					0.27	5.08 ***
Anxiety					0.19	3.58 ***
*R* ^2^	0.43		0.43		0.59	
*F*	44.82 ***		44.61 ***		77.26 ***	

Note: *N* = 1005. IV, independent variable; DV, dependent variable. CRT = Confucian responsibility thinking. Gender: male = 0, female = 1. * *p* < 0.05, ** *p* < 0.01, *** *p* < 0.001.

## Data Availability

The data presented in this study are available on request from the corresponding author.
